# Inferring RBP-Mediated Regulation in Lung Squamous Cell Carcinoma

**DOI:** 10.1371/journal.pone.0155354

**Published:** 2016-05-17

**Authors:** Atefeh Lafzi, Hilal Kazan

**Affiliations:** 1 Department of Health Informatics, Middle East Technical University, Ankara, Turkey; 2 Department of Computer Engineering, Antalya International University, Antalya, Turkey; University of Erlangen-Nuremberg, GERMANY

## Abstract

RNA-binding proteins (RBPs) play key roles in post-transcriptional regulation of mRNAs. Dysregulations in RBP-mediated mechanisms have been found to be associated with many steps of cancer initiation and progression. Despite this, previous studies of gene expression in cancer have ignored the effect of RBPs. To this end, we developed a lasso regression model that predicts gene expression in cancer by incorporating RBP-mediated regulation as well as the effects of other well-studied factors such as copy-number variation, DNA methylation, TFs and miRNAs. As a case study, we applied our model to Lung squamous cell carcinoma (LUSC) data as we found that there are several RBPs differentially expressed in LUSC. Including RBP-mediated regulatory effects in addition to the other features significantly increased the Spearman rank correlation between predicted and measured expression of held-out genes. Using a feature selection procedure that accounts for the adaptive search employed by lasso regularization, we identified the candidate regulators in LUSC. Remarkably, several of these candidate regulators are RBPs. Furthermore, majority of the candidate regulators have been previously found to be associated with lung cancer. To investigate the mechanisms that are controlled by these regulators, we predicted their target gene sets based on our model. We validated the target gene sets by comparing against experimentally verified targets. Our results suggest that the future studies of gene expression in cancer must consider the effect of RBP-mediated regulation.

## Introduction

Aberrant gene expression is a main feature of cancer development. Characterizing the regulatory events that lead to gene expression changes during cancer development is critical for cancer research. Differential gene expression in cancer can occur due to several factors including copy-number variation (CNV), DNA methylation changes, and alterations in transcriptional and post-transcriptional regulatory mechanisms. Among these factors, post-transcriptional regulation (PTR) has gained significant importance due to its emerging roles in cancer biology.

PTR is mediated by the interactions of RNA-binding proteins (RBPs) and microRNAs (miRNAs) with target mRNAs through short sequence and/or structure motifs. Recent studies have found that RBPs are key regulators controlling every step of RNA metabolism including RNA splicing, transport, localization, decay and translation. More than 850 RBPs have been identified in the human genome [1, 2]. Recent advances in experimental methods that characterize the binding sites of RBPs have significantly expanded our knowledge of *in vivo* and *in vitro* RBP binding preferences [3, 4]. This recent explosion of knowledge on RBP binding sites provide opportunities to study RBP-mediated regulation in greater detail.

Several RBPs have been found to be implicated in cancer [5]. For example, overexpression of KHDRBS1 (Sam68) has been revealed in various cancer types including breast, prostate, colorectal and lung cancer cells [6–8]. KHDRBS1 is found to mediate the alternative splicing of oncogenes. ELAVL1 is another well-known RBP that is found to be associated with tumorigenesis by regulating the stability and translation of key growth factors and proto-oncogenes [9, 10]. Overexpression of ELAVL1 has been observed in many cancer types [11, 12]. Recently, FXR1 is found to regulate tumor progression in lung cancer, and is identified as a driver of the 3q amplicon, the most frequent genomic alteration in squamous cell lung cancers [13]. These and many other example indicate that dysregulation of the function or the expression of RBPs has profound implications for cancer development.

Recently developed computational models that study gene expression in cancer have mainly focused on transcriptional regulation and miRNA-mediated regulation. For instance, Setty et al predicted expression changes in glioblastoma (GBM) with a lasso-regularized regression [14]. In addition to CNV and methylation changes, they included features that correspond to TF binding sites from TRANSFAC filtered by DNA hypersensitive regions, and miRNA binding sites obtained from scanning with 7-mer seed sequences. Their model predicted a number of key regulators from TFs and miRNAs that are predictive of survival rate in GBM. Jacobsen et al focused on miRNA-based regulation, ignoring transcriptional-regulation [15]. They looked at the relation between the expression of miRNAs and mRNAs in tumors from 11 human cancer types in TCGA, and identified a pan-cancer miRNA-mRNA network. Li et al proposed a two-stage regression framework that combines data from TCGA and ENCODE to predict gene expression in Acute Myeloid Leukemia (AML) [16]. Their model revealed a number of TFs and miRNAs as candidate regulators of AML. To the best of our knowledge, there is still no study that investigates the effects of RBP-mediated regulation in cancer.

In this study, we propose to explain gene expression in cancer with a statistical model that incorporates RBP-based regulation in addition to CNV, DNA methylation and the regulatory effects of transcription factors and miRNAs. As a case study, we applied our model to Lung squamous cell carcinoma (LUSC) dataset, as we found that there are a large number of differentially expressed RBPs in this cancer type. By comparing the performance of our full model with partial models that exclude one of the feature groups (e.g. TFs, CNV etc.), we show that the added predictive value of RBPs is higher than all the other feature groups. Following up on this result, we applied a feature selection procedure to identify the RBPs as well as other factors that play a key role in LUSC. Importantly, majority of our predicted candidate regulators are previously found to be associated with lung cancer, and are differentially expressed. Subsequently, we determined the targets of these candidate regulators, and compared against experimentally determined targets. The results of this study suggest that future studies of gene regulation must consider the effects of RBP-mediated regulation.

## Materials and Methods

### Data integration and preprocessing

All mRNA and miRNA expression data were obtained from TCGA data portal [17]. For regression analysis, scaled estimate column from RNA-seq (level 3) datasets were downloaded. Scaled estimate data were multiplied by 10^6^ to obtain relative abundance (in Transcripts Per Million [TPM]). Genes that have ≤ 0.1 TPM in more than 70% of the samples were removed. TPM values were log2 transformed for subsequent analysis. For miRNA expression, Illumina HighSeq (387 samples) and Illlumina GA (136 samples) datasets were combined. GISTIC2-processed DNA copy-number data and DNA methylation (Level 4) data were retrieved from Firehose (http://gdac.broadinstitute.org/runs/analyses__2014_10_17)). For each gene, the methylation probe that shows the strongest negative correlation (Pearson correlation coefficient) between methylation “Beta-value” and mRNA expression across all samples was selected. We used Human Proteome Atlas to determine the RBPs and TFs that are expressed in lung cancer [18]. Human Proteome Atlas provides immunohistochemistry results on 12 tumors for each cancer type [19]. RBPs and TFs that show expression in at least one of these tumors were considered as expressed.

### Differential expression analysis

We downloaded datasets for cancer types that have sufficient number of paired samples (i.e., >15 matched tumor-normal samples). According to this criteria, the following 13 cancer types were selected: BLCA, BRCA, COAD, HNSC, KICH, KIRC, KIRP, LIHC, LUAD, LUSC, PRAD, THCA and UCEC (S1 Table). We downloaded raw counts from RNA-seq (level 3) datasets. We filtered out the genes that are not expressed in the majority of the samples by removing those genes with ≤ 1 read count per million in more than 50% of samples [20]. We used edgeR to calculate the log fold changes (LFCs) of genes that code for RBPs within each cancer type [21]. In particular, we determined differential expression using the generalized linear model likelihood ratio test (using *glmFit* and *glmLRT* functions). We defined differentially expressed RBPs as those genes with FDR cutoff < 0.05 and |*LFC*| > 0.5. We plotted the LFCs of RBPs across the cancers as a heat map and performed row and column clustering using *heatmap.2* function in R (hierarchical clustering performed with Pearson correlation distance and average linkage). We performed a similar analysis to identify differentially expressed miRNAs after filtering miRNAs that have less than 1 read count per million miRNA reads in more than 70% of samples.

### Target prediction of regulatory elements

#### Transcription factors

Promoters were defined as the ±2000 bp region around the transcription start sites (TSS) of genes based on Refseq annotation. Position frequency matrices (PFMs) of human transcription factors (for 382 TFs) were obtained from JASPAR database [22]. We used the motif scanning tool FIMO from MEME-Suite [23] to map the binding sites of transcription factors along the promoter regions (selected matches with p-value < 1*e* − 4). Next, we counted the sites that intersect with DNaseI hypersensitive regions determined in A549 cells [24] to define the feature vectors of TFs.

#### MicroRNAs

We downloaded the human 3’UTRs compiled by Agarwal et al [25]. These 3’UTRs were derived by extending Gencode annotations (Harrow et al., 2012) with recent data on 3’UTR isoforms [26]. To define the miRNA target sites, we downloaded conserved targets from the recently released TargetScan database (v7, [25]). The input vector for a miRNA feature includes the counts of target sites on each gene.

#### RNA-binding proteins

To determine the targets of RBPs, we downloaded the PFMs of 85 human RBPs from the RNAcompete paper [4]. We identified RBP sites by scanning human 3’UTRs with the top 10 n-mers generated by these PFMs. Moreover, we downloaded the motifs for the following well-known RBPs from RBPDB database [27]: HNRNPAB, PUM1, PUM2, ELAVL2, KHSRP, ZFP36, AUF1 and CUGBP. Sites of these RBPs were determined similarly. We downloaded CLIP-seq and PARCLIP data for a list of RBPs (ELAVL1, FMR1, FUS, FXR1, FXR2, hnRNPC, IGF2BP1-3, LIN28, PTBP1, PUM2, QKI, SRSF1, TIA1) from starBase database [28]. In addition to these RBP specific CLIP datasets, we downloaded gPARCLIP-determined peaks that correspond to regions occupied by any of the expressed RBPs in HEK293 cells [1]. We intersected CLIP- and gPARCLIP-determined peaks with human 3’UTRs. To account for background binding bias in CLIP-based techniques (identified in [29]) we excluded the parts of peaks that overlap with regions that correspond to background binding. Next, we determined the RBP sites that are located in these peaks.

### LUSC dataset

After compiling all the datasets for LUSC, we first intersected the CNV, DNA methylation and binding datasets of TFs, RBPs and miRNAs. This resulted in 12,436 genes and 362 tumor samples in common. Then, we removed those regulatory factors that have no binding site in any of these genes. Finally, we merged the features that have identical input vectors (i.e., identical counts across the genes) to a single feature (S2 Table). In the end, we had 204 TFs, 164 miRNAs, 49 RBPs as our regulatory features.

### Feature selection

In order to determine candidate regulators in LUSC, we performed a feature selection procedure specifically developed for lasso-regularized regression models. We downloaded the Selective Inference package from R and used the *fixedLassoInf* function to calculate selective p-values for a given lambda (i.e. regularization constant) value. Here, we used the lambda that is selected with *cv.glmnet* function. We repeated this procedure for each sample independently, and calculated, for each feature, the number of times a significant p-value (p-val < 0.05) is obtained.

### Identification of target gene sets of candidate regulators

We summed the changes in prediction error of each gene across the samples when a regulator is removed. To estimate the significance of the error changes, we repeated this calculation with shuffled feature matrices 5000 times [14]. The shuffling was done for each column independently. We calculated an empirical p-value (for a gene-regulator pair) by comparing the error change obtained from the original feature matrix with the distribution of error changes that are obtained from shuffled feature matrices. The target gene set of a regulator is defined as the genes with FDR-corrected p-value <= 2*e* − 4. We compared the predicted target genes of RBPs against CLIP-based targets. To evaluate the predicted targets of miRNAs, we downloaded experimentally verified targets from MirTarBase database [30]. We grouped the experimentally verified targets based on the type of evidence. Namely, targets identified with reporter assay, western blot and qPCR provide strong evidence; whereas targets identified with approaches such as microarray, NGS-based methods, pSILAC provide less strong or weak evidence.

## Results

### Differentially expressed RBPs in cancer

**Fig 1:**
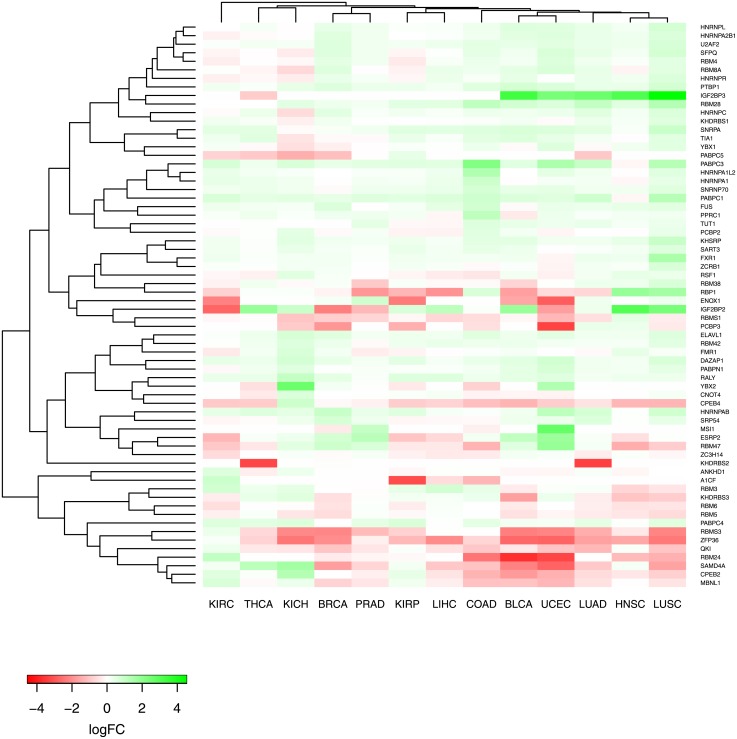
Differentially expressed RBPs in cancer. This heatmap shows the log fold expression changes of RBPs across matched tumor-normal samples (calculated with edgeR [21]). Rows are the RBPs that are differentially expressed in at least one cancer type. Columns correspond to different cancer types. Rows and columns are clustered with hierarchical clustering.

Cancer is commonly characterized by the differential expression of several master regulators. In particular, aberrant expression of RBPs have been found to be associated with cancer initiation and progression [31]. To investigate the expression changes of RBPs in cancer systematically, we downloaded matched tumor-normal samples for 13 cancer types (see S1 Table for a summary of these datasets). We used edgeR to identify differentially expressed genes across the matched samples for each cancer (see S3 Table for results). Fig 1 shows the log fold changes (LFCs) of RBPs that are differentially expressed in at least one of these cancer types (LFC > 0.5 or LFC < −0.5, FDR-corrected p-value < 0.05). We observed that a number of well-known RBPs (e.g. PTBP1, KHSRP, ELAVL1, PABPC1, PABPC3, HNRNPAB) display increased expression in majority of cancer types. Among these RBPs, ELAVL1 has been previously found to have elevated levels of expression in cancer [32]. On the other hand, RBPs such as CPEB4, RBMS3, QKI and ZFP36 show decreased expression across the majority of cancer types. Interestingly, FXR1 is found to be overexpressed most in LUSC compared to the other cancer types. Indeed, a recent study revealed FXR1 as a driver for non-small cell lung cancer (NSCLC), and showed that increased FXR1 promotes tumor progression, and is associated with poor survival [13]. Lastly, we observed that IGF2BP2 and IGF2BP3 display strong up- or down-regulation of expression among the different cancer types. In particular, both IGF2BP2 and IGF2BP3 are overexpressed significantly in LUSC. Proteins that belong to the IGF2BP family are known to be expressed mainly in the embryo; however, they have been found to be re-expressed in several cancer types including lung cancer [33]. We also observe interesting patterns when we cluster the cancer types based on LFCs of RBPs. Interestingly, LUSC is found to be more similar to head and neck squamous cell carcinoma compared to lung adenocarcinoma indicating the importance of cell type of origin. In parallel with our observation, these three cancer types have been previously assigned to a single Pan-Cancer subtype in terms of protein expression [34]. Another sub cluster is formed from bladder urothelial carcinoma and uterine corpus endometrial carcinoma, and the high similarity of these two cancer types in terms of protein expression has been also previously observed [34].

### Predicting gene expression in cancer

**Fig 2:**
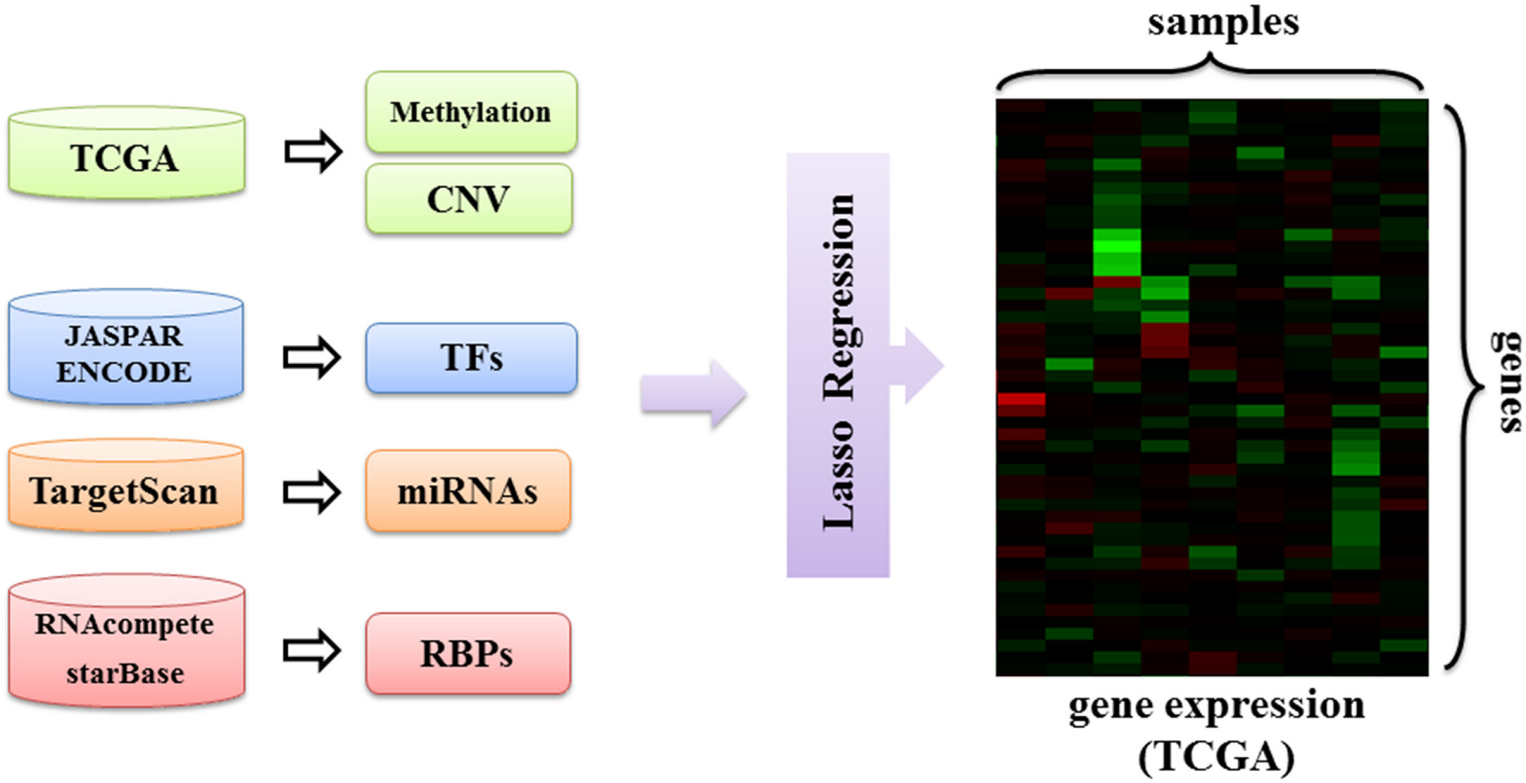
Overview of the proposed regression model. DNA methylation, copy number variation and regulatory effects of transcription factors, miRNAs and RBPs are input to a lasso regularized regression model to predict gene expression in LUSC.

Having found that many RBPs are differentially expressed in LUSC, we set out to investigate the regulatory effects of RBPs in this cancer type in more detail. To this end, we developed a regression model that incorporates copy number variation, DNA methylation and the regulatory effects of transcription factors, miRNAs and RBPs as features to predict gene expression in LUSC (Fig 2):

$$y_{g}=w_{0}+w_{C}C_{g}+w_{M}M_{g}+\sum_{TF}w_{TF}N_{g}^{TF}+\sum_{miR}w_{miR}N_{g}^{miR}+\sum_{RBP}w_{RBP}N_{g}^{RBP}$$

where *y*_*g*_ is the expression of gene *g*, *C*_*g*_ is the CNV of gene *g*, *M*_*g*_ is the gene’s methylation level and $N_{g}^{TF}$, $N_{g}^{miR}$ and $N_{g}^{RBP}$ are the counts of binding sites of TFs, miRNAs and RBPs for gene *g*, respectively. As there are a large number of parameters in this model, we also included a lasso regularization term to enforce sparsity. In this way, we aimed to find a small set of features that best explain gene expression. We used the glmnet package [35] to learn the model where we set the regularization constant using cross-validation (CV).

### Performance evaluation

**Fig 3:**
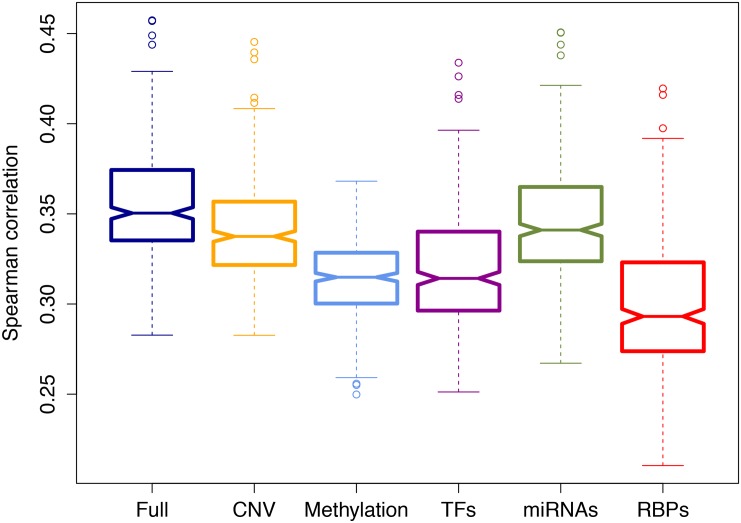
Added predictive value of regulator types. This box plot displays the Spearman rank correlation between the predicted and the actual held-out genes in 10-fold cross- validation (CV) averaged across all the samples. The full model that uses all features was compared with the partial models that lack one of the regulator groups: CNV (copy number variation), DM (DNA methylation), TFs, miRNAs, RBPs.

To evaluate the performance of the model, we fit a regression model for each sample separately, and performed 10-fold CV. For each CV run, we calculated the Spearman rank correlation between predicted and observed expression of genes in the held-out set. We then averaged these correlation values across the CV folds, and then across the samples. When we used all the features described above, we obtained a Spearman rank correlation of 0.36. To determine the predictive value of features, we compared the full model with partial models that exclude one of the regulatory classes. Fig 3 shows how average Spearman rank correlation changes when one type of regulatory class (i.e., CNV, DM, TFs, miRNAs and RBPs) is removed from the model. This comparison revealed that RBPs show the greatest added predictive value (16% reduction when omitted) followed by TFs (10% reduction) and methylation (10% reduction). CNV (4% reduction) and miRNAs (3% reduction) contribute relatively less to the predictive performance (S4 Table). The strong association of methylation and TFs with gene expression have been previously observed several times, whereas the remarkably high effect of RBP-mediated regulation in explaining gene expression in cancer is a novel result.

### Candidate regulators of LUSC

Having found that regulatory factors can explain a significant portion of gene expression in LUSC, we used a feature selection procedure to determine the predominant regulators. A common approach to test the importance of an additional feature between two nested linear models is to compare the change in error to a chi-square distribution or F-distribution. However, this approach becomes invalid when the additional feature is chosen adaptively as in lasso regularized regression [36]. Despite this fact, F-test has been previously used for lasso [16]. Here, we improve over previous studies by applying a significance test (i.e., covariance test) that accounts for adaptivity [37]. We applied the covariance test for each sample independently, and counted the number of times a significant p-value (p-value < 0.05) is obtained for each feature. Table 1 shows the regulatory factors that are selected in more than 60 samples. In addition to the name and type of the regulator, the log fold change and the associated FDR-corrected p-value are also displayed if the regulator is found to be differentially expressed (complete list of candidate regulators is available in S5 Table). Among the candidate regulators, LIN28A is ranked the first as it was selected as a significant predictor in 40% of all samples. LIN28A is an evolutionarily conserved RBP that is known to increase proliferation by inhibiting let-7 biogenesis [38]. The next significant predictors are DNA methylation (38% of all samples) and copy-number variation (37% of all samples). ELAVL1, which ranks fourth in our list of candidate regulators, has key functions in mRNA stability and translation. In fact, cytoplasmic ELAVL1 expression has been previously found to be associated with high tumor grade and poor survival rate in non-small cell lung carcinoma [39]. Indeed, we found that ELAVL1 is upregulated in LUSC (LFC = 0.59). We see several other RBPs that are ranked on top of this list. For instance, SFPQ, which is selected as a significant regulator in a large number of samples, has been recently found to interact with a long non-coding RNA called MALAT1 (metastasis-associated lung adenocarcinoma transcript 1) [40]. MALAT1 is overexpressed in several human cancers including non-small cell lung cancer, and has been identified as a critical regulator of metastasis in lung cancer cells [41, 42]. YY1 (Ying Yang 1) is the top ranking regulator among the TFs (LFC = 0.83). YY1 is highly expressed in various cancer types, and its depletion inhibits tumor formation of breast cancer cells [43, 44]. We found that miR-1 is the top ranking miRNA regulator. A recent study revealed that miR-1 was significantly reduced in lung squamous cell carcinoma, and its restoration significantly reduced cancer cell progression [45]. The second ranking miRNA, miR-218, is significantly down regulated in lung squamous cell carcinoma and has been identified as candidate tumor suppressor [46]. The LFCs that are calculated with our differential expression analysis are in agreement with these studies (LFCs -3.47 and -2.09 for miR-1 and miR-218 respectively). Lastly, though located on the lower part of the list, FXR1 is one of the identified candidate regulators. A previous study has identified FXR1 as a key regulator of tumor progression and found that its overexpression is critical for nonsmall cell lung cancer (NSCLC) cell growth [13]. Similarly, we found that FXR1 is significantly upregulated in LUSC (LFC is 1.49). In summary, this table reveals that many of the candidate regulators are differentially expressed between cancer and normal samples. Altogether, the high correspondence between our predicted candidate regulators and previous literature indicates that our model is accurate in inferring the key regulators of LUSC.

**Table 1:** Selected candidate regulators.

Regulator	Type	Selection %	logFC	p.value
LIN28A	RBP	40	-	-
Met	TF	38	-	-
CNV	TF	37	-	-
ELAVL1	RBP	35	0.59	9.65e-16
YY1	TF	32	0.83	1.06e-29
SFPQ	RBP	30	0.77	3.41e-14
PABPN1	RBP	29	0.47	7.2e-09
miR-1	miRNA	28	-3.47	3.15e-37
ZC3H14	RBP	27	-0.14	0.084
miR-218	miRNA	27	-2.09	3.7e-31
miR-142-3	miRNA	26	0.42	0.027
miR-153	miRNA	25	0.26	0.437
let-7e	miRNA	25	-0.45	0.05
HNRNPC	RBP	25	0.64	7.33e-13
RBM6	RBP	24	-0.45	1.35e-08
G3BP2	RBP	21	0.02	0.856
NRF1	TF	21	0	0.966
RBMS1	RBP	20	-0.33	0.09
miR-494	miRNA	20	0.32	0.428
REST	TF	19	0.39	0.06
CPEB4	RBP	19	-1.3	7.68e-25
miR-1224	miRNA	19	0.47	0.283
CDX1	TF	19	-	-
miR-143	miRNA	18	-1.62	1.07e-19
ETV6	TF	18	0.52	1.23e-05
miR-375	miRNA	18	-2.69	1.5e-14
PABPC4	RBP	18	0.56	2.13e-08
RBM4	RBP	18	0.48	8.12e-11
miR-421	miRNA	17	0.67	0.01
GLIS1	TF	17	0	0
SART3	RBP	17	0.58	9.27e-15

### Target analysis of candidate regulators

**Fig 4:**
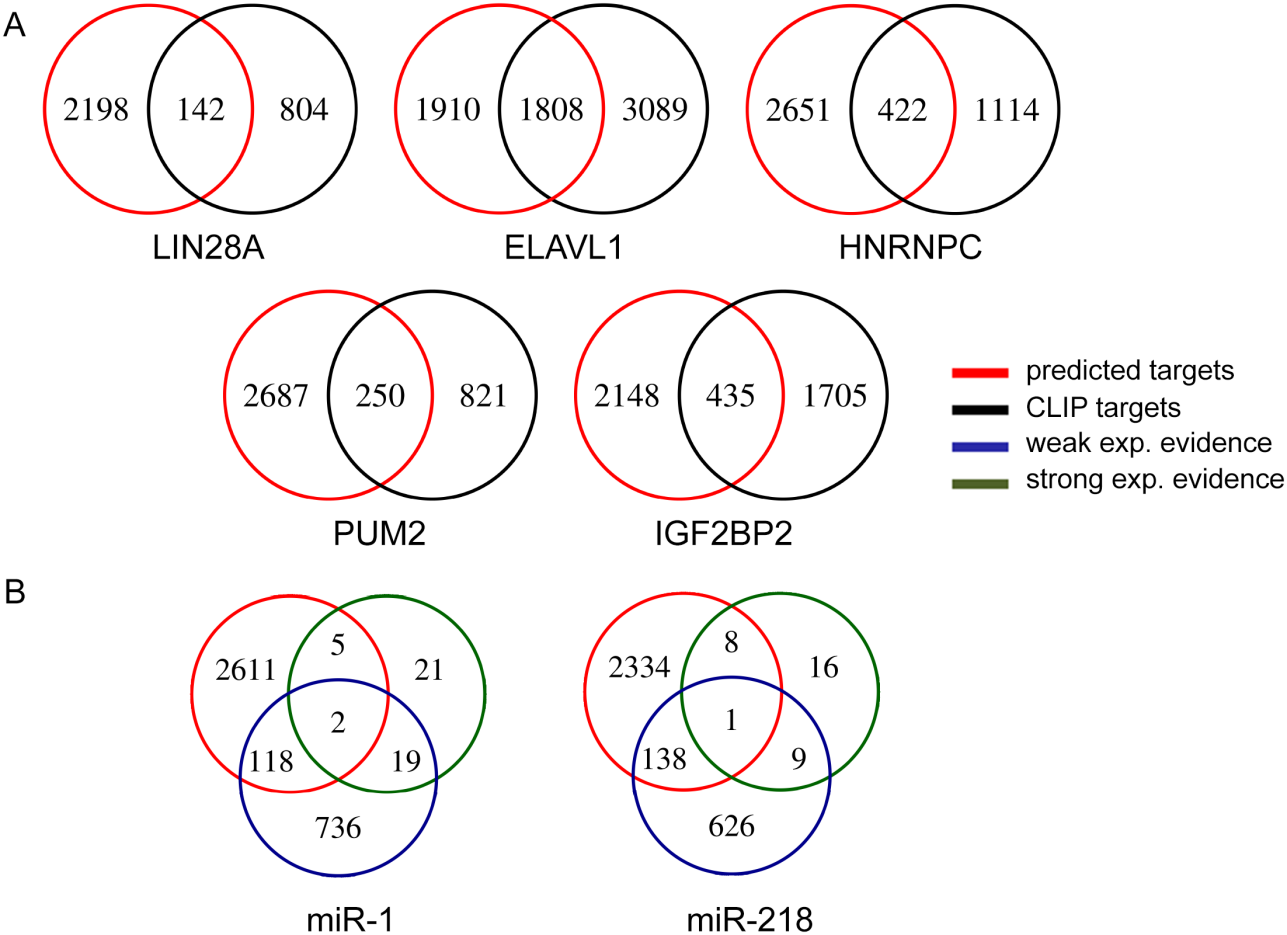
Intersection of predicted target genes with experimentally verified targets. A. Predicted target gene sets of RBPs are intersected with targets determined with CLIP method. B. Predicted targets of miRNAs are intersected with experimentally verified (with either weak or strong evidence) miRNA targets downloaded from miRTarBase.

The input feature matrix that we compiled by counting the number of binding sites of each regulator in each gene provides a noisy approximation of functional targets of regulators. To identify the targets of the regulators from our model robustly, we identified the genes for which the squared prediction error increases when a regulator is removed. We determined the significance of an increase in error by comparing it against a distribution of error changes that are obtained when the feature matrix is randomized (Materials and Methods). We evaluated our predicted target gene sets by comparing against experimentally verified interactions, when available. For RBPs, our validation set consists of the genes that are identified by CLIP experiment. As such, we could evaluate the target sets of RBPs with CLIP data: LIN28A, ELAVL1, HNRNPC, PUM2 and IGF2BP2. We evaluated the target predictions for our top ranking miRNAs miR-1 and miR-218, by compiling experimentally verified targets (either with strong evidence or weak evidence) from MirTarBase database. Fig 4 shows the number of genes that are shared between the set of our predicted targets and the set of experimentally verified targets, for RBPs and miRNAs. We see a high overlap between the two sets for RBPs. In particular, almost 50% of the predicted target genes for ELAVL1 are also CLIP targets. The intersection is much smaller for miRNAs. A similar result has been previously obtained when miRNA target prediction methods were compared based on the number of validated targets in miRTarBase [16].

**Fig 5:**
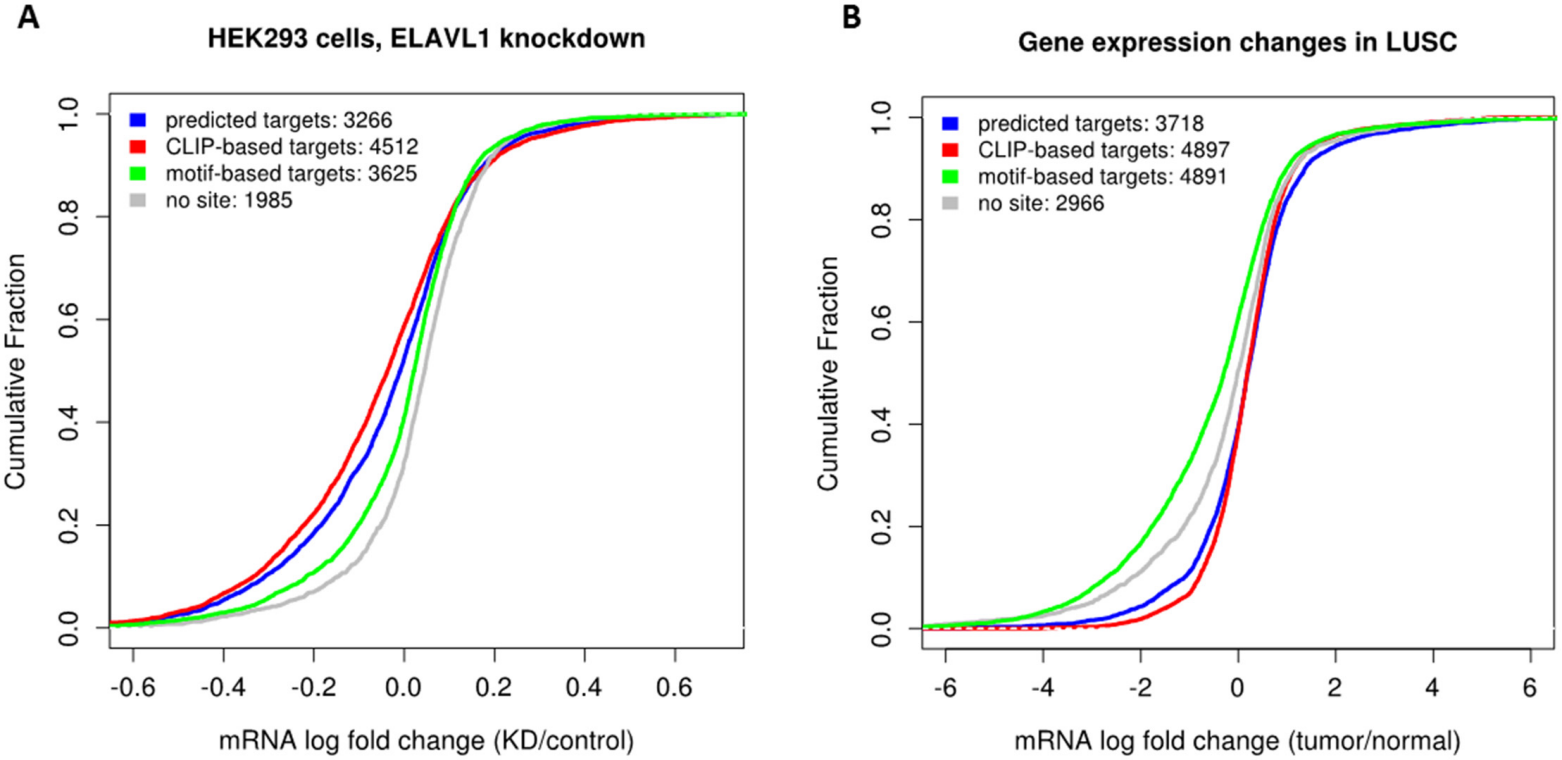
Evaluation of predicted target genes of ELAVL1. A. Cumulative distribution of LFCs of genes upon ELAVL1 depletion in HEK293 cells. B. Cumulative distribution of LFCs that correspond to gene expression changes in tumor samples compared to normal samples.

Next, we utilized a previously published ELAVL1 knockdown dataset that includes genome-wide measurements of transcripts upon ELAVL1 depletion in HEK293 cells [47]. ELAVL1 is known to increase the stability of its targets, so we expect ELAVL1 targets to have decreased expression upon its depletion. In Fig 5A we plot the cumulative distribution of LFCs of the following groups of genes: (i) CLIP-derived target genes, (ii) target genes predicted based on our model (*predicted targets*), (iii) genes that have at least one ELAVL1 motif but that do not appear in groups (i) and (ii) (*motif-derived targets*), (iv) genes with no ELAVL1 motif (*no site*). This analysis revealed that our predicted targets have lower LFCs than motif-derived targets and genes with no ELAVL1 site indicating that our predicted target genes are likely to be functional targets. Furthermore, we made a similar comparison between these groups based on expression changes of genes across matched tumor-normal samples. Namely, we plot the cumulative distribution of LFCs calculated with edgeR (Fig 5B). We observe that the distributions of LFCs of our predicted target genes and CLIP-derived target genes are close. Since ELAVL1 is upregulated in LUSC (LFC = 0.59), we expect its targets to be upregulated as well. In line with this, our predicted target genes have larger LFCs than the groups (iii) and (iv). Altogether these results support the accuracy of our model in identifying the functional targets of ELAVL1.

## Discussion

In this study, we investigated the mechanisms that account for gene expression regulation in LUSC. We initially assessed the alterations in expression of genes that encode for RBPs across a number of cancer types. To our knowledge, this is the first time differentially expressed RBPs are searched using a method that accounts for matched samples across several cancer types (i.e. edgeR). The results of this analysis revealed that several RBPs are differentially expressed with district profiles of up- or down-regulation across the cancers. Having found that the number of differentially expressed RBPs is largest in LUSC, we developed a lasso-regularized regression model to predict gene expression in LUSC by incorporating several features including the regulation mediated by RBPs. We were able to accurately predict the expression of genes in held-out sets by incorporating a comprehensive set of regulatory elements that are bound by TFs, miRNAs and RBPs, as well as genetic and epigenetic alterations as features in our statistical model. Importantly, compared to other regulatory classes, exclusion of RBPs results in the largest decrease in predictive performance revealing the influence of RBP-mediated regulation. This is one of the key novel observations of the current study that indicates the importance of RBPs in regulation gene expression in LUSC.

Next, we identified key regulators of LUSC by calculating the significance of each feature using a recently proposed statistical test that accounts for the adaptive nature of fitting lasso models. Inference of statistically significant features in adaptive models is an active research area in statistics, and we believe that our study will be instrumental in dissemination of this recent result to bioinformatics community. We found that the majority of the top ranking candidate regulators are differentially expressed in LUSC, and have been previously identified to be associated with lung cancer. We have also identified additional regulators such as LIN28A and CPEBP4 that were not previously studied in the context of lung cancer. Also, the fact that many of the candidate regulators are RBPs agrees with our previous result on the added predictive value of RBPs.

As a follow-up analysis, we utilized our fitted model parameters to identify target genes of candidate regulators. We evaluated our predicted target gene sets by comparing against experimentally identified targets. We found that nearly ∼ 50% of our predicted ELAVL1 targets overlap with CLIP-derived target genes. This overlap was relatively smaller for the other regulators. A reason for this observation could be the fact that these experiments were performed in other cell types than lung cancer cells. Furthermore, CLIP protocol and the experimental techniques that miRTarBase database considers only aim to identify whether the regulator of interest physically binds to an mRNA. As such, not all the target genes identified by these techniques may correspond to functional targets. Our further analysis of ELAVL1 targets by utilizing a genome-wide ELAVL1 knockdown dataset allows us to investigate the functional relationship between ELAVL1 and our predicted targets.

Apart from the results on RBP regulation, our study is also amongst the first to incorporate the recently released JASPAR and TargetScan databases in predicting TF and miRNA binding sites, respectively. Identification of TF and miRNA target sites can become more accurate with the availability of ChiP-Seq and CLIP-seq datasets in lung cells. Similarly, CLIP experiments have been performed for a small number of RBPs, and increase in the number of such experiments would improve the definition of RBP target sets. Another possible improvement would be the consideration of alternative polyadenylation (APA) in defining RBP and miRNA sites. Recently developed computational models such as DaPars [48] and GETUTR [49] can perform de novo identification of APA events from standard RNA-seq data. These methods can be used to determine the 3’UTR isoform distribution for each sample (i.e., tumor) separately. Subsequently, RBP and miRNA sites can be defined for each sample uniquely based on this distribution. Furthermore, RNA secondary structure, which is an important factor for target recognition of some RBPs, has been ignored in the current study. RNA secondary structure can be considered in the identification of RBP binding sites as more RBPs have characterized secondary structure preferences. Also, recent advances in experimental techniques to query secondary structure *in vivo* [50, 51] promise to generate a more accurate set of mRNA secondary structures compared to the computational prediction methods.

In our model, we made a simplifying assumption that TFs, RBPs and miRNAs can bind to mRNA independently. However, multiple TFs can bind to the same promoter in a competitive or collaborative fashion. Similarly, recent studies show that RBPs and miRNAs can act in competition or collaboration with each other [52]. Increased knowledge on these interactions will be instrumental in developing more accurate models of regulatory networks in the future.

Lung cancer is one of the most difficult cancers to treat. Recently developed molecular therapies can be targeted to adenocarcinoma of the lung [53]. Such a treatment has not been proposed for squamous cell carcinoma yet. Therefore, identification of novel therapeutic agents is vital for this cancer type. Here, we applied our novel statistical model to infer gene regulatory mechanisms in LUSC, and identified a number of candidate regulators including RBPs. Further studies of these candidate regulators will provide insights into the molecular mechanisms of cancer development in LUSC.

## Supporting Information

S1 Table Summary of analyzed TCGA cancer types and datasets. This table lists the abbreviations and full names of the analyzed cancer types together with the corresponding number of total and paired samples downloaded from TCGA database.
(PDF)

S2 Table List of the features that are merged. Features that have identical count vector across the genes have been merged to a single factor before input to the regression.
(XLSX)

S3 Table Differential expression of RBPs across the 13 cancer types. This table contains the log fold changes and the corresponding FDR-corrected p-values for RBPs (plotted in Fig 2).
(XLSX)

S4 Table Comparison of models. This table displays the Spearman correlation coefficient of the full model and the five partial models where one feature group is removed. P-values indicate the significance of the difference between models (Wilcoxon sign-rank test).
(PDF)

S5 Table List of all candidate regulators. Full list of the names, types and LFCs of candidate regulators are displayed.
(XLSX)
