# Supplementary material for: Inferring RBP-Mediated Regulation in Lung Squamous Cell Carcinoma
Source: PLoS One. 2016 May 17;11(5):e0155354. doi: 10.1371/journal.pone.0155354 (PMC4871487; doi:10.1371/journal.pone.0155354)
Supplement: S1 Table — This table lists the abbreviations and full names of the analyzed cancer types together with the corresponding number of total and paired samples downloaded from TCGA database. (PDF) [file pone.0155354.s001.pdf]

Table S1: Summary of analyzed TCGA cancer types and datasets			
Cancer type	Description	All samples	Paired samples
BLCA	Bladder urothelial carcinoma	408	19
BRCA	Breast invasive carcinoma	1095	113
COAD	Colon adenocarcinoma	459	41
HNSC	Head and neck squamous cell carcinoma	521	43
KICH	Kidney chromophobe	66	25
KIRC	Kidney renal clear cell carcinoma	533	72
KIRP	Kidney renal papillary cell carcinoma	290	32
LIHC	Liver hepatocellular carcinoma	371	50
LUAD	Lung adenocarcinoma	516	58
LUSC	Lung squamous cell carcinoma	501	51
PRAD	Prostate adenocarcinoma	497	52
THCA	Thyroid carcinoma	505	59
UCEC	Uterine corpus endometrial carcinoma	545	23
