# Supplementary material for: Inferring RBP-Mediated Regulation in Lung Squamous Cell Carcinoma
Source: PLoS One. 2016 May 17;11(5):e0155354. doi: 10.1371/journal.pone.0155354 (PMC4871487; doi:10.1371/journal.pone.0155354)
Supplement: S4 Table — This table displays the Spearman correlation coefficient of the full model and the five partial models where one feature group is removed. P-values indicate the significance of the difference between models (Wilcoxon sign-rank test). (PDF) [file pone.0155354.s004.pdf]

Table S4: Comparison of models

	Mean Spearman correlation	Median Spearman correlation	p-value (compared to Full model)
Full	0,355	0,35	N/A
CNV excluded	0,34	0,337	7,69E-61
Methylation excluded	0,313	0,315	4,44E-61
TFs excluded	0,319	0,314	4,44E-61
miRNAs excluded	0,344	0,341	4,44E-61
RBPs excluded	0,297	0,293	4,44E-61
